# Concurrent Complementary and Alternative Medicine CAM and Conventional Rehabilitation Therapy in the Management of Children with Developmental Disorders

**DOI:** 10.1155/2013/812054

**Published:** 2013-11-12

**Authors:** Soo Yeon Kim, Yong-Il Shin, Sang-Ook Nam, Chang-Hyung Lee, Yong Beom Shin, Hyun-Yoon Ko, Young-Ju Yun

**Affiliations:** ^1^Department of Rehabilitation Medicine, Pusan National University Yangsan Hospital, Pusan National University School of Medicine, Yangsan, Republic of Korea; ^2^Research Institute for Convergence of Biomedical Science and Technology, Pusan National University Yangsan Hospital, Yangsan, Republic of Korea; ^3^Department of Pediatrics, Pusan National University Yangsan Hospital, Pusan National University School of Medicine, Yangsan, Republic of Korea; ^4^Department of Rehabilitation Medicine, Pusan National University Hospital, Pusan National University, School of Medicine, Busan, Republic of Korea; ^5^Department of Integrative Medicine, School of Korean Medicine, Pusan National University Yangsan Hospital, Yangsan, Republic of Korea

## Abstract

*Background*. We investigated the concurrent use of conventional rehabilitations and complementary and alternative medicine (CAM) therapies for the long-term management of children with developmental disorders (DDs). *Methods*. The parents or caregivers of 533 children with DDs (age range, 1–19 years) who visited the rehabilitation centers were surveyed using in depth face-to-face interviews. *Results*. Of the 533 patients enrolled, 520 completed the questionnaire (97% response rate). A total of 292 (56%) children were receiving multiple therapies, more than two conventional rehabilitations and CAM, at the time of the interview. A total of 249 (48%) children reported lifetime CAM use, 23% used CAM at the time of the interview, and 62% of the patients planned to use CAM therapy in the future. Conventional rehabilitation therapies used at the time of the interview included physical therapy (30%), speech therapy (28%), and occupational therapy (19%), and the CAM therapies included herbal medicine (5%) and acupuncture or moxibustion (3%). The respondents indicated that in the future they planned to use acupuncture or moxibustion (57%), occupational therapy (18%), cognitive behavioral therapy (16%), speech therapy (10%), and physical therapy (8%). *Conclusion*. Concurrent management as conventional rehabilitations and CAM therapies is widely used by children with DDs.

## 1. Introduction

Children with developmental disorders (DDs) do not achieve developmental milestones in motor, perceptual, speech, cognition, and behavioral areas. DDs are relatively common with an estimated prevalence of 3~4% worldwide [1]. Children with DDs are at risk of chronic physical, developmental, behavioral, and emotional conditions that need special treatment in addition to that required by healthy children. Early intervention has been shown to improve developmental outcomes and reduce the socioeconomic impact of DDs [1]; thus, children with DDs need a wide range of treatments. 

Children with DDs often receive a combination of long-term conventional rehabilitation therapies, such as physical therapy, occupational therapy, speech therapy, counseling, and cognitive behavioral therapy, in multiple rehabilitation centers. The use of complementary and alternative medicine (CAM) to treat children is on the rise in developed countries with a prevalence ranging from 1.8% to 80%, depending on the population and study design [2–6]. A recent study reported that 36% of general pediatric patients and 61.9% of children with epilepsy used CAM in USA [7]. CAM includes homeopathy, phytotherapy, acupuncture, moxibustion, anthroposophic medicine, and Bach flower remedies.

Oriental medicine is considered a type of CAM by Western medicine practitioners [8]. CAM is an important form of healthcare in Korea and is an alternative treatment option for patients with DDs. Few studies have examined the concurrent use of conventional rehabilitation programs provided in multiple clinical settings and CAM to manage DDs. In acknowledgment of the increasing public interest in CAM, we assessed the motivation, prevalence of use (if ever used after the diagnosis of a DD), and perceived effectiveness of long-term conventional multicenter and CAM therapies in Korean patients with DDs.

## 2. Materials and Methods

Between March and August 2011, 533 children with DDs (age range, 1–19 years) from two hospitals in Korea were enrolled in the present study. We conducted face-to-face interviews with 533 parents or caregivers of the children about the use of the conventional rehabilitation therapies in multiple centers and CAM. The questionnaire was designed using previous data [3, 9–13], list of CAM and conventional rehabilitation therapies, and input from practitioners and was reviewed by a panel of experts. One researcher conducted all interviews taking care not to express personal views about conventional rehabilitation and CAM therapies. In addition, each patient underwent a 15 min questionnaire-based interview conducted by a trained nurse in the hospitals' outpatient clinics. The questionnaire addressed four areas: children's characteristics (age, sex, diagnosis, and disease duration), concurrent management at the time of the survey, those in the past, and future plans for therapy. Concurrent management was defined as concurrent CAM and conventional rehabilitation therapy. Statistical tests were conducted using the Statistical Package for Social Sciences version 15 (SPSS Inc., Chicago, IL, USA), frequency analysis, and chi-square test. The study was approved by the local ethics committee.

## 3. Results

### 3.1. Demographic Characteristics

Of the 533 patients enrolled, 520 completed the questionnaire (97% response rate). Of those, 296 were males and 224 were females. Mean age was 7.5 ± 2.3 years. The diagnoses were epilepsy, 368 (71%), cerebral palsy, 70 (13%), attention deficit hyperactivity disorder, 33 (6%), and other such as mood disorders or musculoskeletal problems, 49 (10%). The demographic characteristics of the survey respondents are summarized in Table 1.

### 3.2. Management Strategies

A total of 292 (56%) children were receiving concurrent management at the time of the interview, and 25% had previously undergone concurrent management, but they were not receiving it at the time of the interview. Concurrent management used at the time of the interview was physical therapy (30%), speech therapy (28%), occupational therapy (19%), counseling or cognitive behavioral therapy (8%), herbal medicine (5%), acupuncture or moxibustion (3%), mineral and vitamin therapy (13%), and manual therapies (0.4%). 

Treatments used more than once in the past were physical therapy (8%), occupational therapy (9%), speech therapy (8%), counseling or cognitive behavioral therapy (3%), herbal medicine (13%), acupuncture or moxibustion (11%), mineral and vitamin therapy (6%), and manual therapies (3%). Multiple responses were permitted for each question. 

**Figure 1:**
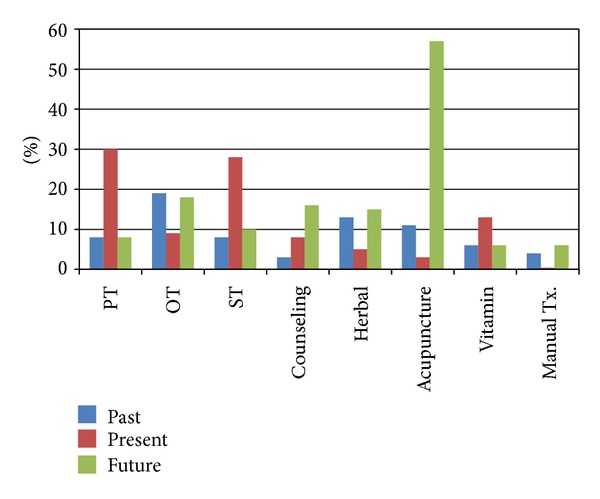
Types of rehabilitation treatment used by children with developmental disorders. Of 520 children with developmental disorders, 292 (56%) used multiple conventional rehabilitation and CAM therapies administered in multiple centers. PT, physical therapy; OT, occupational therapy; ST, speech therapy.

Finally, we assessed the need for continued therapy in the future. The results showed that 62% of the parents or caregivers were considering future treatment such as acupuncture or moxibustion (57%), herbal medicine (7%), mineral and vitamin therapy (6%), manual therapies (6%), occupational therapy (18%), cognitive behavioral therapy (16%), speech therapy (10%), and physical therapy (8%) (Figure 1). 

There were no significant differences of concurrent management between boys and girls. Seventy-three percent of concurrent management users at present plan to use CAM in the future. There were significant differences for planning CAM in the future between concurrent management users and nonconcurrent management users statistically (*P* < 0.05). 

### 3.3. Reasons for Discontinuing CAM or Conventional Rehabilitation Medicine

We found that 34% of the children had used CAM, and 23% were using CAM at the time of the interview. Moreover, 26% of the children had used conventional rehabilitation therapy in the past, and 47% were using conventional rehabilitation therapy at the time of the interview. The reasons given for discontinuing CAM were improved condition, 39%; noneffectiveness, 36%; patient did not cooperate, 14%; financial burden, 13%; inconvenience (distance or time), 13%; and side effects, 9%. The reasons given for discontinuing conventional rehabilitation therapy included inconvenience (distance or time), 33%; noneffectiveness, 17%; improved condition, 11%; financial burden, 11%; patient did not cooperate, 10%; and side effects, 2%.

## 4. Discussion

Various conditions can cause developmental delays, and children with DDs exhibit several clinical symptoms that may require long-term management; thus, a range of therapeutic options are necessary for these patients. Because of its chronic nature, physical medicine and rehabilitation are the cornerstones of DD treatment. Generally, children with DDs undergo more than one type of therapy in multiple rehabilitation centers. However, a significant proportion of patients use, and frequently perceive a benefit from, alternative medicine [14], and CAM therapies are widely used by pediatric patients with DDs in Korea. The reasons parents and caregivers gave for choosing CAM therapies included positive views of the complementary therapies, limitations of conventional rehabilitation medicines, concern about the adverse effects of conventional medicine, doctors' recommendation, and the increased availability of complementary therapies [15–17]. In Korea, Traditional Oriental Medicine is performed by certified Korean oriental medical doctors; thus, patients tend to view CAM as more acceptable and natural than conventional medicine. The recent growing interest in CAM has stimulated publications on CAM treatment for various medical conditions [17]. In response to the increasing public interest in CAM, our survey assessed the expectations and prevalence of CAM among patients with DDs and their parents or caregivers.

Current estimates based on previous studies indicate that 19% to 57% of patients with DDs use CAM [3, 9–11]. In Korea, 54% of stroke patients [12] and 82% of patients with rheumatoid arthritis [13] have been reported to use CAM. In fact, the rate of CAM use is higher in people with poor health, chronic conditions, and/or a low functional status than in healthy people [18]. 

The present study found that 292 (56%) children with DDs used concurrent conventional rehabilitation and CAM therapies administered in multiple settings. For example, patients may have enrolled in similar physical therapy programs in multiple rehabilitation centers, possibly because the parents/caregivers believed that the various rehabilitation facilities had different strong points. However, in using multiple centers, the patients risk high costs and inconsistent care across centers.

**Figure 2:**
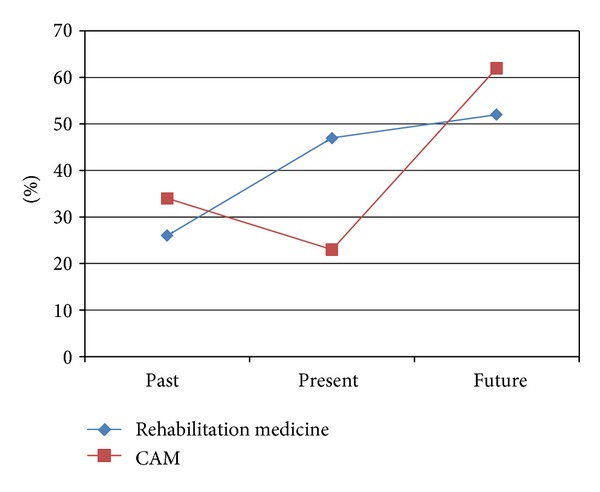
Trends in concurrent management for children with developmental disorders. Of 520 children with development disorders, 249 (48%) reported lifetime CAM use, and 23% were using CAM at the time of the interview. Furthermore, 62% of the patients planned to use CAM management in the future.

A total of 249 (48%) of the children reported lifetime CAM use, 23% were using CAM at the time of the interview, and 62% of the patients planned to use CAM treatments in the future (Figure 2). Of the various CAM therapies, 57% of patients planned to use acupuncture and moxibustion for future management. More higher percent of patients in concurrent management users plan to use CAM in the future compared to nonconcurrent management users. Our study showed wide use of, and an increasing demand for, CAM therapies. 

The most common reasons for discontinuing CAM treatment were improved condition, noneffectiveness, and side effects, whereas inconvenience was the most common reason why patients discontinued conventional rehabilitation therapies.

The appropriate use of CAM involves engaging a medical doctor and oriental medical doctor and ensuring good communication between both doctors and the patient. Medical doctors must recognize that complementary medicines are widely used by patients with DDs and be aware of potential adverse effects. Physicians should determine which CAM therapies their patients use and advise them and their families about the evidence-based efficacy and side effects of the treatments. Intervention by the physician may be necessary because concurrent CAM and conventional rehabilitation therapies can inadvertently cause side effects or render the treatment ineffective. The wide range of CAM treatments used by the families of children with DDs in our study highlights the ongoing search for effective and balanced treatment for these lifelong conditions. The pediatric medical community needs to be aware of CAM use to provide better education, understanding, and optimal patient-physician relationships.

The present study is the first to investigate the use of concurrent conventional rehabilitation medicine and CAM for the management of children with DDs in Korea. We identified the types of CAM used by patients with DDs, those most commonly used, and reasons for discontinuing CAM.

Furthermore, we investigated the management of DDs using concurrent conventional rehabilitation therapies with overlapping programs in multiple centers and CAM.

The limitations of this study are cross-sectional design and being unable to make any conclusions about causality. This study reveals high percentage of caregivers who plan to use CAM in the future. Although one researcher conducted the interviews and did not express personal views about CAM or conventional rehabilitation therapies, just asking caregivers about these therapies could influence answers given. Participants were enrolled from only two hospitals in Korea; thus, the findings are not generalizable to all Korean children with DDs and limited to other children with DDs in other countries. However, our findings reveal trends in therapy preferences for children with DDs in Korea. 

## 5. Conclusion

The present survey showed that a higher percentage of Korean children with DDs currently use multiple conventional rehabilitation therapies administered in overlapping programs; however, we found increasing interest in using CAM for future treatment. Our findings suggest that medical practitioners need to be aware of the various types of conventional and CAM therapies to provide comprehensive, integrated medical services for children with DDs. 

## Figures and Tables

**Table 1:** Characteristics of children with developmental disorder.

Characteristics	
Age (year), mean ± standard deviation	7.5 ± 2.3
Sex, *n* (%)	
Male	296 (57%)
Female	224 (43%)
Diagnosis, *n* (%)	
Epilepsy	368 (71%)
Cerebral palsy	70 (13%)
ADHD	33 (6%)
Others	52 (10%)
Disease durations (year), mean ± standard deviation	5 ± 1.9

ADHD: attention deficit hyperactivity disorder.
